# BioSift: A Dataset for Filtering Biomedical Abstracts for Drug Repurposing and Clinical Meta-Analysis

**DOI:** 10.1145/3539618.3591897

**Published:** 2023-07-18

**Authors:** David Kartchner, Irfan Al-Hussaini, Haydn Turner, Jennifer Deng, Shubham Lohiya, Prasanth Bathala, Cassie Mitchell

**Affiliations:** Georgia Institute of Technology, Atlanta, Georgia, USA; Georgia Institute of Technology, Atlanta, Georgia, USA; Georgia Institute of Technology, Atlanta, Georgia, USA; Georgia Institute of Technology, Atlanta, Georgia, USA; Georgia Institute of Technology, Atlanta, Georgia, USA; Georgia Institute of Technology, Atlanta, Georgia, USA; Georgia Institute of Technology, Atlanta, Georgia, USA

**Keywords:** drug repurposing, document filtering, active learning, weak supervision

## Abstract

This work presents a new, original document classification dataset, BioSift, to expedite the initial selection and labeling of studies for drug repurposing. The dataset consists of 10,000 human-annotated abstracts from scientific articles in PubMed. Each abstract is labeled with up to eight attributes necessary to perform meta-analysis utilizing the popular patient-intervention-comparator-outcome (PICO) method: has human subjects, is clinical trial/cohort, has population size, has target disease, has study drug, has comparator group, has a quantitative outcome, and an “aggregate” label. Each abstract was annotated by 3 different annotators (i.e., biomedical students) and randomly sampled abstracts were reviewed by senior annotators to ensure quality. Data statistics such as reviewer agreement, label co-occurrence, and confidence are shown. Robust benchmark results illustrate neither PubMed advanced filters nor state-of-the-art document classification schemes (e.g., active learning, weak supervision, full supervision) can efficiently replace human annotation. In short, BioSift is a pivotal but challenging document classification task to expedite drug repurposing. The full annotated dataset is publicly available and enables research development of algorithms for document classification that enhance drug repurposing.

## 1 INTRODUCTION: DRUG REPURPOSING VIA NATURAL LANGUAGE PROCESSING

The development of clinical drugs is an expensive process requiring billions of dollars in research and development to bring a new drug to market [7, 46, 55]. Drug repurposing seeks to reduce the cost of discovering new treatments by identifying currently approved drugs with therapeutic value for other diseases [2]. Doing so relies on aggregating clinical studies and data to identify therapeutic combinations of the highest value [3].

Drug repurposing (sometimes called drug repositioning) is the use of an existing drug for a different disease or indication other than the one for which it was initially developed or marketed [39]. Drug repurposing is a safe and cost-effective way to expedite treatment discovery. It is particularly effective for novel, rare, or intractable diseases where current standard-of-care treatments are inadequate. For example, repurposed drugs were critical during the initial onset of the SAR-CoV-2 (COVID-19) pandemic [40]. Even if a repurposed drug may not fully ameliorate a new disease, it could be a powerful adjuvant therapy that enhances the efficacy of existing standard-of-care treatments or decreases adverse events or side effects. Drug repurposing may be done by evaluating molecular similarities; comparing shared biochemical targets; examining associations with adverse event profiles; examining the effect of popular therapeutics for common antecedent diseases or co-morbidities; or other forms of measured association between a drug and a specific patient attribute. Once a repurposed drug candidate is identified, it can undergo expedited clinical testing due to the existing safety profiles. If the repurposed drug candidate is deemed successful, it may undergo standard regulatory approval for the new indication or be prescribed off-label if the new indication is too rare for a standard clinical trial.

Searching, filtering, reviewing, and analyzing large volumes of scientific literature is critical to the drug repurposing process. On average, 75+ clinical trials are published each day [6]. Traditional efforts to synthesize data from the literature for drug repurposing, systematic review, or meta-analysis primarily rely upon PubMed advanced search filters to index and retrieve candidate documents. Unfortunately, neither standard nor advanced PubMed search filters enable efficient filtering of critical attributes for meta-analysis. Typically only a very small proportion of retrieved PubMed documents meet inclusion criteria [12, 38] for meta-analysis. Document filtering remains a pivotal bottleneck in drug repurposing meta-analysis [1]. Improved automatic document filtering is needed to remove irrelevant documents and improve downstream processes for curating data necessary for drug repurposing.

To this end, we construct and release an extensive annotated data set, BioSift, that enables improved filtering based on attributes utilized for meta-analysis in drug repurposing. Namely, most meta-analyses employ the patient-intervention-comparator-outcome or *PICO* method when determining if a document has the elements necessary for study inclusion: **P**: What are the patient population and quantitative sample size? I: What is the defined intervention or study drug? **C**: Is there a comparator population, and how is it defined? **O**: What is the quantitative clinical outcome? BioSift consists of 10,000 biomedical abstracts labeled with up to eight attributes necessary to perform meta-analysis for drug repurposing: has human subjects, is clinical trial/cohort study, has population size, has target disease, has study drug, has comparator group, has a quantitative outcome, and an “aggregate” label. Each abstract was annotated by 3+ different annotators (i.e., biomedical students), and a sample was reviewed for quality/correction by senior quality control.

Experiments demonstrate that our dataset enables more nuanced document inclusion/exclusion than is available in PubMed advanced search alone. BioSift enables users to screen out 70+% of returned articles not containing relevant data. Thus, BioSift significantly decreases the research time required for filtering articles for biomedical evidence synthesis. Current results illustrate that current active learning, weak supervision, and full supervision algorithms are not able to fully automate the filtering process for drug repurposing. However, BioSift is an extremely valuable open resource for continued machine learning development of improved document filtering algorithms for drug repurposing.

This paper makes the following contributions:

- We develop a protocol for filtering documents relevant to drug discovery using defined attributes that better emulate the PICO review process utilized by clinical scientists.
- We present a human-annotated dataset of 10,000 PubMed abstracts with eight unique filtering attributes or labels than indicate an article’s likely utility for inclusion in a clinical meta-analysis.
- We present three low-resource and one fully-supervised baseline to compare different automated strategies for biomedical abstract filtering in the absence of annotation resources.

## 2 DATASET

**Figure 1:**
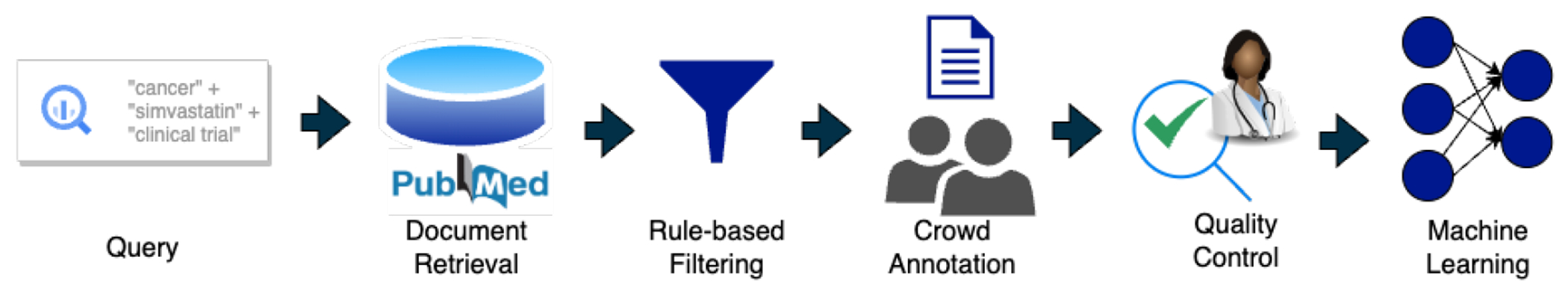
Overall annotation pipeline

**Figure 2:**
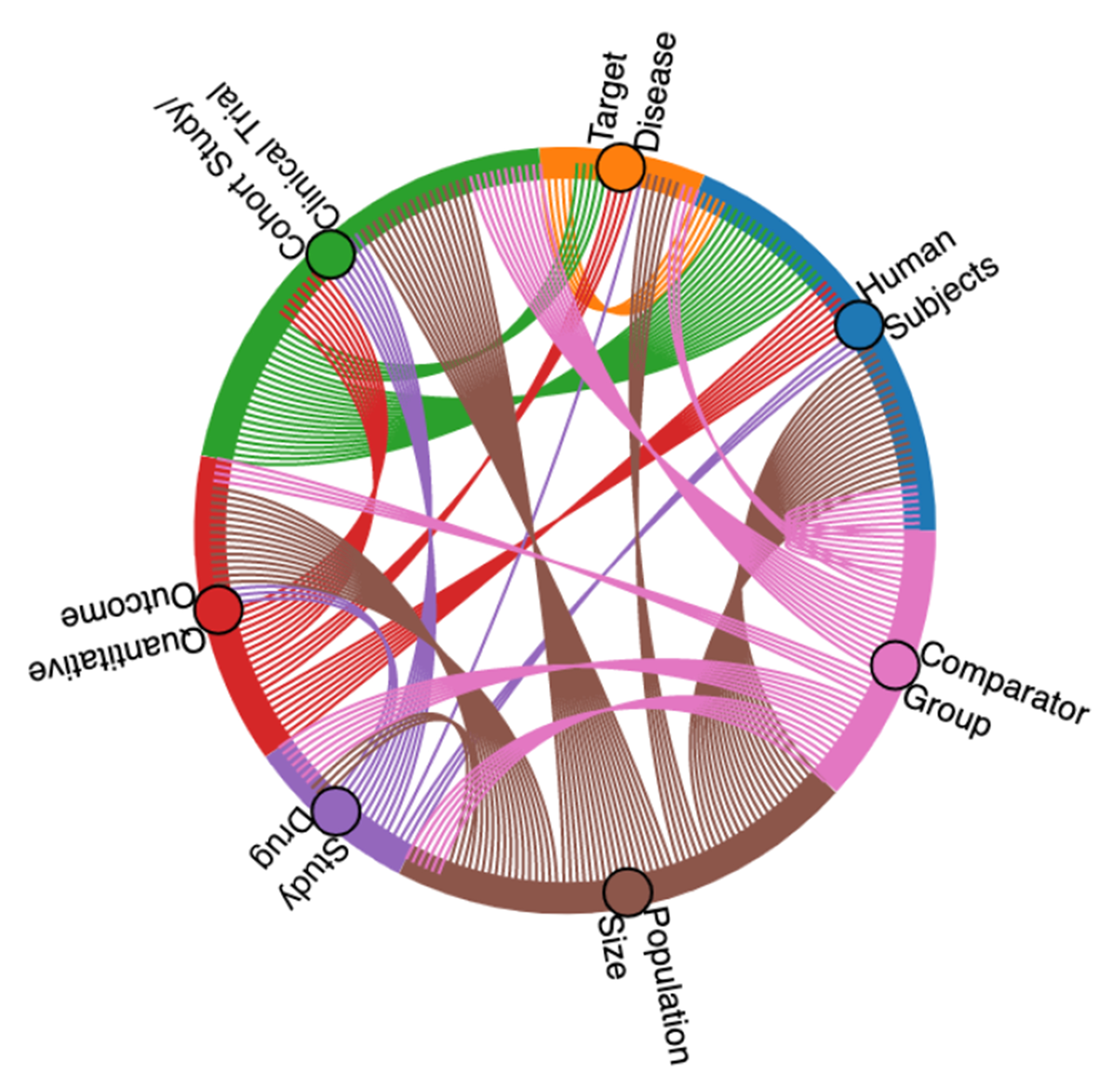
Chord Co-occurrence Diagram

We present, BioSift, a collection of 10,000 documents labeled with multiple criteria to filter clinical studies containing relevant information for drug repurposing. Inclusion criteria were chosen based on collaboration with epidemiological experts to retain only abstracts containing sufficient information to be used in a meta-analysis on drug repurposing potential. Inclusion criteria and other document statistics are shown in Table 1. Three or more curators annotated each document, with expert curators checking a sample of disagreeing labels during a quality control phase. A depiction of the end-to-end document selection, filtering, and annotation process is shown in Figure 1, and the relative co-occurrence of the seven labels in BioSift is shown in Figure 2.

### 2.1 Document Selection

Candidate documents for annotation were selected from PubMed to target research abstracts focused on drug repurposing for cancer Initial PubMed queries were designed to include only cohort studies and clinical trials satisfying at least one of two inclusion criteria:

- Abstract addresses at least one type of cancer.
- Abstract includes at least one treatment for common cancer comorbidities or antecedent diseases such as diabetes, hypertension, asthma, hypothyroidism, sleep disorders, neuropathy, hyperlipidemia, depression, etc.

We performed preliminary document filtering by creating a pool of documents from PubMed queries of the form “*cancertype*” AND (neoplasm OR cancer OR tumour)) OR “*cancertype*”[MeSH]) AND (“*drug*_1_” OR “*drug*_1_”[MeSH] OR “*drug*_2_” OR “*drug*_3_” OR …) AND (“clinical trial” OR “retrospective” OR “prospective” OR “case control” OR “case-control”), where entities *cancertype* and $drug_{i}$ are replaced with names and/or Medical Subject Headings (MeSH) titles of cancer types and drug respectively. The objective of this query was to gather clinical evidence of whether drugs used to treat comorbidities or antecedent diseases had a positive or negative effect on cancer outcomes. The pool of documents was taken as the union of results for these queries for 8 different types of cancer and 94 non-cancer drugs. Following a PubMed search, abstracts were further filtered by removing those that did not have any chemical entities in their MeSH terms or had 5 or fewer words in the text of the abstract. The final post-filtering pool of documents contained 58,720 unique abstracts, from which we randomly selected 10,000 for annotation.

### 2.2 Annotator Selection and Training

The dataset was annotated by a cohort of 58 university undergraduate students selected from biology, computer science, neuroscience, and biomedical engineering majors. Additionally, 10 students with prior annotation training and experience were recruited as quality control managers. The BioSift student annotation program was similar to our previous award-winning undergraduate biocuration program [41].

The annotator recruiting process consisted of two rounds of screening. First, a graded assessment was used to evaluate the candidates’ untrained “annotation aptitude” using a simplified schema similar to the present study. Candidates who achieved a satisfactory score were interviewed in small groups (less than 6 students). Candidates were asked a series of questions regarding their interest in the project and their problem-solving strategies. Of the 83 candidates who applied for the position, 58 were ultimately recruited as Biosift annotators.

Annotator training was conducted over a 6-week period. First, students participated in live lectures designed to introduce them to the annotation schema, annotation software, relevant vocabulary, and context surrounding the project goals. Next, students were given formal annotation training, including annotation guides and worked examples that defined the labeling schema, live guidance in labeling practice abstracts, self-paced practice annotation problems, and graded practice annotation assessments.

Prior to annotating BioSift, a 2-week beta test was performed to assess the developed schema and the success of the annotator training. At the conclusion of the beta test, annotators were surveyed for feedback regarding the study label schema and annotation platform. Beta test results were used to refine the training resources and final BioSift labeling schema to reduce error and improve inter-annotator agreement.

During all stages (training, beta test, and final annotation of BioSift) the students were given tools to openly communicate directly with each other, the quality control managers, and research coordinators via an electronic communication platform and live virtual discussions.

### 2.3 Final Annotation and Data Quality Control

Each abstract in BioSift was annotated by 3+ different students using LightTag [45]. The annotators were encouraged to submit comments with challenging or confusing abstracts to proactively prevent errors due to semantic or lexical misunderstandings. All curated abstracts without inter-annotator disagreement and without comments were accepted without manager-level quality control. If there was inter-annotator disagreement, the abstract was reviewed by a separate quality control manager to correct the abstract’s annotations.

Quality control (QC) for BioSift data was conducted by a team of 10 student managers with both formal annotation training and at least 6 months of previous annotation experience. The quality control team was directly involved in training the student annotators and creating annotation resources for the project. The managers received additional quality control training from the research study coordinator. The quality control protocol required the managers: 1) to validate and/or fix potential annotation errors; 2) review and resolve inter-annotator disagreement to discern a final “ground truth” annotation for each abstract.

The final round of quality control involved ranking the articles in descending order of disagreement levels between the three annotators across the seven classes. The articles with the highest disagreement levels were assigned a final round of quality control with two annotators for each article. First, confidence level of each annotator was ranked based on the agreement with the ground truth labels for a gold set of 25 articles. QC annotations with the complete agreement were taken as ground truth. For QC annotations with disagreement, the final label was determined as the annotation of the annotator with higher confidence score. The statistics and results in this paper pre-date this final round of quality control which affects < 1% of annotations. The data incorporating this quality control will be available in the GitHub repository.

### 2.4 Dataset and Annotation Statistics

**Figure 3:**
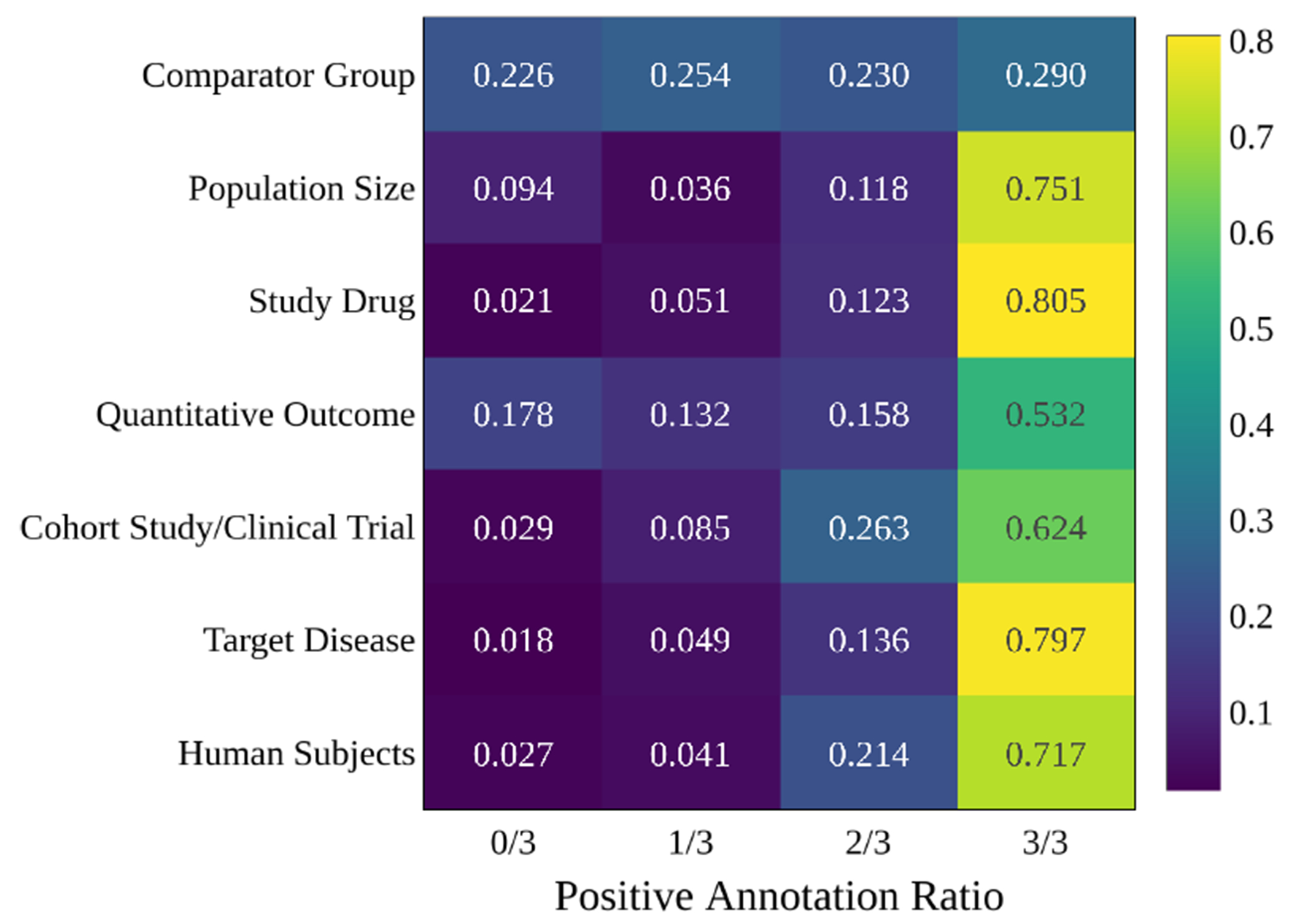
Inter-Annotator Agreement for Each Class

For the 10,000 annotated abstracts in BioSift, we evaluate the positive annotation ratio for each label class, inter-annotator agreement, and co-occurrence between positive label schema. Figure 3 shows the proportion of inter-annotator agreement for each class. It demonstrates that more than 50% of all labels except Comparator Group are annotated with positive labels by all three annotators.

**Figure 4:**
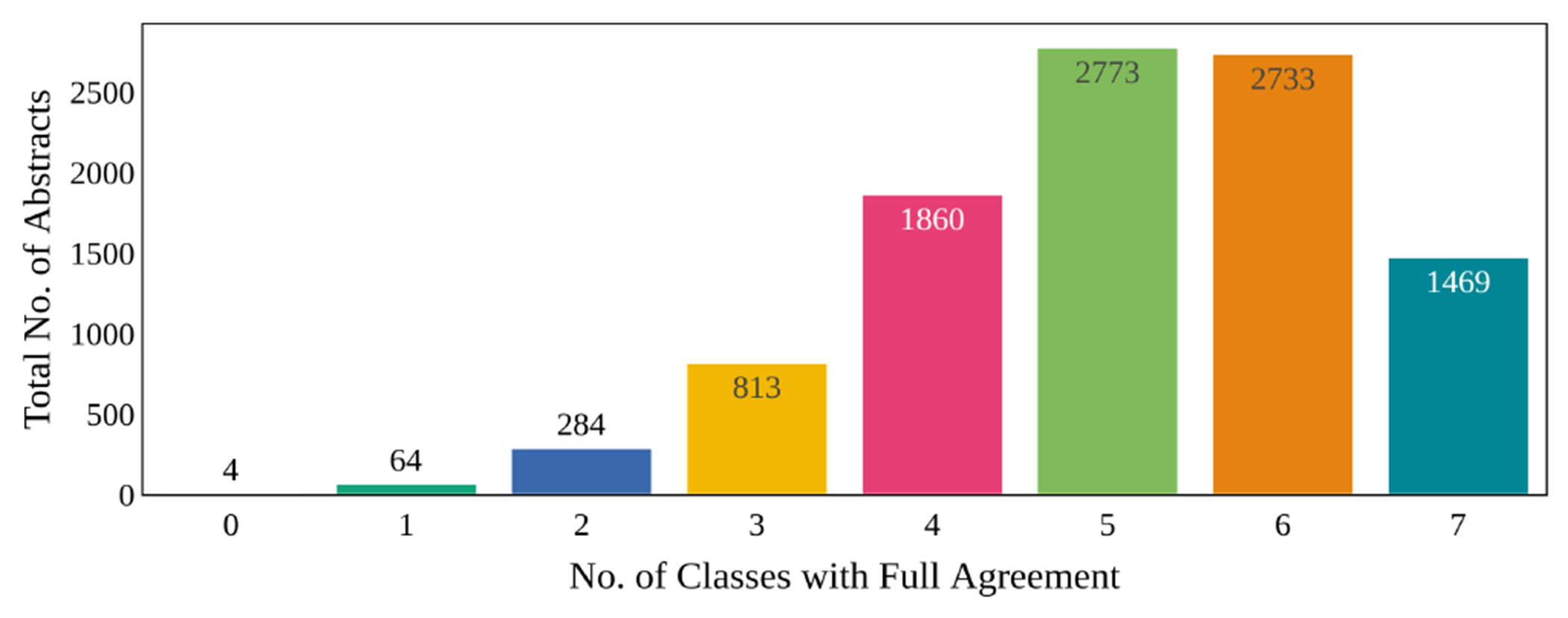
Count Agreement

Figure 4 shows the distribution of the number of labels with complete agreement among annotators. It shows that 4 or more labels are in complete agreement in most abstracts.

**Figure 5:**
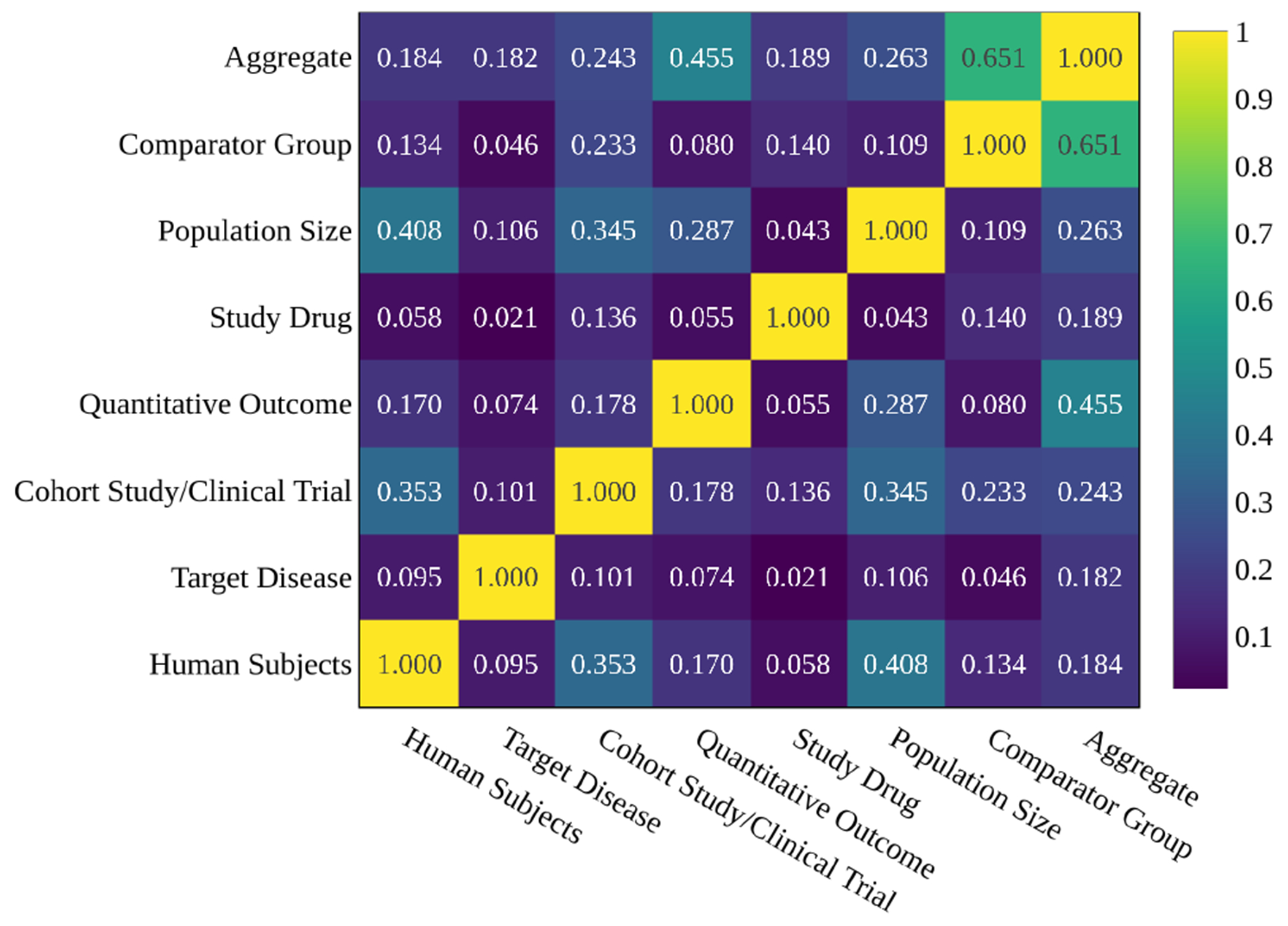
Co-occurrence and aggregated effect

We define the annotation ratio $=\frac{\text{Number of positive annotations}}{\text{Total number of annotations}}$ and assign each category a positive binary label when the annotation ratio exceeds 0.6. The aggregate label for an abstract is positive when all category labels are positive. Figure 5 shows the Pearson correlation coefficient between the binary labels, including the aggregate label. It highlights that some labels are strongly correlated, like Population Size with Quantitative Outcome, Human Subjects, and Cohort Study/Clinical Trial. It also shows that the Quantitative Outcome and Comparator Group have the most significant effect on the aggregate label.

**Figure 6:**
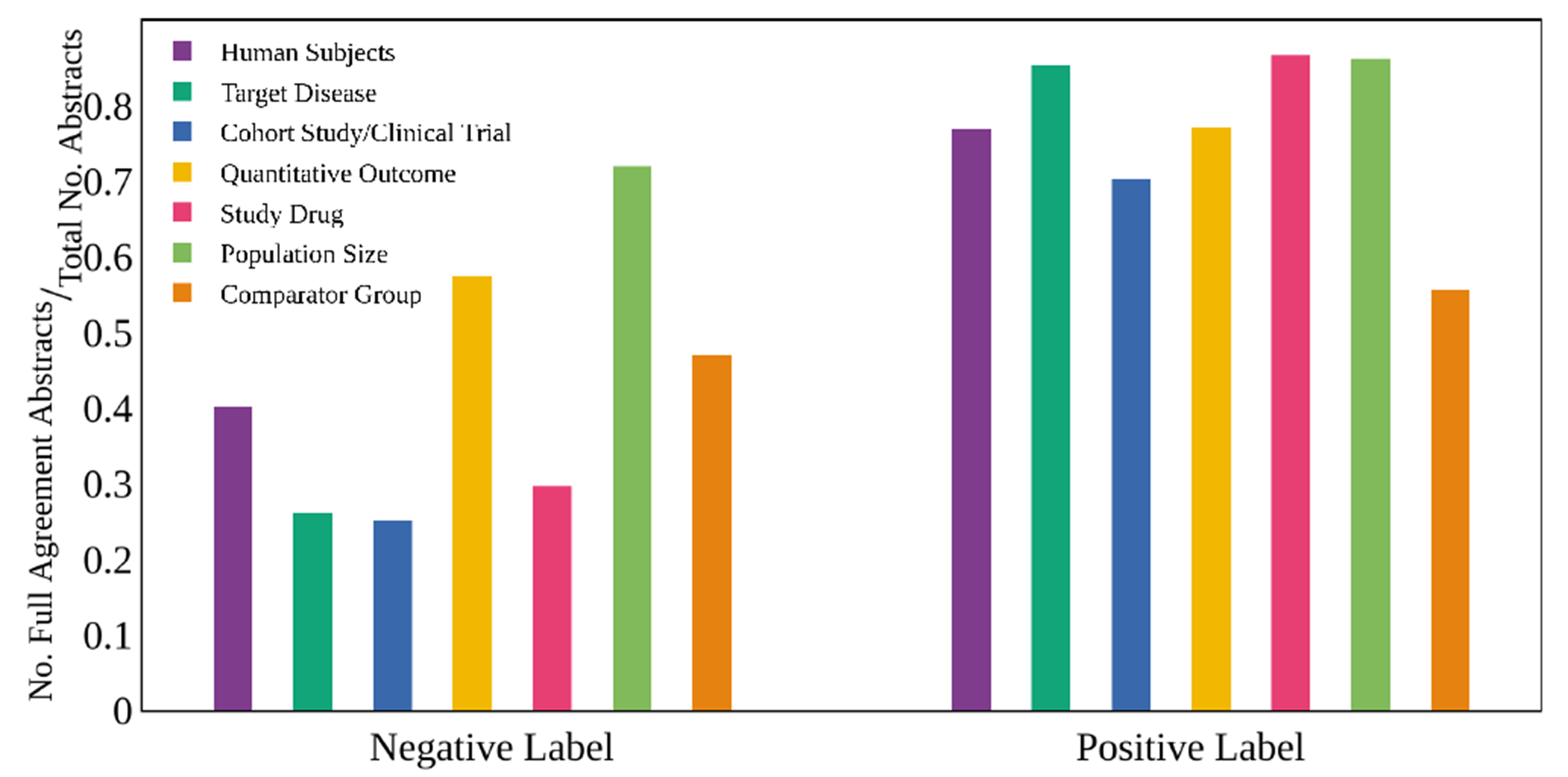
Inter-annotator Agreement

We additionally observe that positive labels have higher interannotator agreement than negative labels, pictured in Figure 6.

## 3 METHODS

The document filtering/classification task presented in BioSift is one that has normally been solved by carefully crafted queries (e.g., Cochrane Highly Sensitive Search [11]), supplemented with post-filtering based on rules, heuristics, and machine learning models [1, 37, 38, 53]. Since manual curation resources are often very limited due to the high cost of obtaining reviewers with sufficient medical expertise, previous work has primarily relied upon machine learning methods that generalize well with little to no labeled data. We accordingly test a slate of models taken from active learning, weak supervision, and prompt-based zero-shot learning domains and compare them to fully-supervised transformer models fine-tuned on our data. We additionally compare these models with results from carefully crafted PubMed advanced search queries. Results illustrate that document filtering for drug repurposing meta-analysis is a difficult task and that utilization of BioSift data meaningfully improves document filtering.

### 3.1 Problem Formulation

We formulate the document filtering problem in BioSift as a multilabel classification task with 7 independent labels + a binary aggregate label as described in section 2. For each class, we report the precision, recall, and F1-score of each evaluated model, defined as:

(1) $$\mathrm{Precision}=\frac{TP}{TP+FP}$$

(2) $$\mathrm{Recall}=\frac{TP}{TP+FN}$$

(3) $$F1=\frac{2*\mathrm{Precision}*\mathrm{Recall}}{\mathrm{Precision}+\mathrm{Recall}}$$

where *TP*, *FP*, and *FN* and the counts of true-positives, falsepositives, and false-negatives, respectively.

### 3.2 Weakly Supervised Learning

Weak supervision is the use of programmatic labeling to obtain noisy estimates of labels on data points. Programmatic labeling functions (LFs) generally take the form of heuristics, expert-defined rules, lookups in dictionaries/databases, or outputs of other models used to approximate labels for a given task. Since weak supervision does not rely on ground truth labels, labeling functions can be applied to both labeled and unlabeled documents to create a larger pool of training documents than would otherwise be possible.

For our document filtering task, we develop (LFs) comprised of keyword rules, regular expressions, and NER models to identify evidence of each inclusion criterion. Rules were written with the software package Snorkel [47] with LF outputs defined as ABSTAIN = −1; EXCLUDE = ∅; INCLUDE = 1. For categories where it is difficult to craft rules that can precisely exclude documents (e.g., *Has comparator group, Has population size*), ABSTAIN labels were labeled as EXCLUDE as done in [12] to avoid excessive LF imbalance. We created a total of 32 LFs which collectively matched 99.1% of the instances in our dataset. A comprehensive list of LFs grouped by inclusion criterion can be found in Table 8.

The LFs were used to generate weak labels for the entire labeled BioSift corpus as well as the remaining 46,720 unlabeled documents. For each inclusion criterion, LF outputs were aggregated by majority voting (MV) to form a higher-confidence weak label for the document. We also tried aggregating weak labels with the generative label model described in [47] but found that it produced inferior results to MV. Aggregated weak labels were used to fine-tune a pre-trained biomedical language model to allow prediction on documents unmatched by some or all LFs. The model was fine-tuned using masked binary cross entropy (BCE) loss:

(4) $$H_{p}(q{)}^{\prime }=-\frac{1}{N}\sum_{i=1}^{N}1_{y\neq -1}\left(y_{i}\cdot \log\left(p\left(y_{i}\right)+\left(1-y_{i}\right)\cdot \log\left(1-p\left(y_{i}\right)\right)\right)\right.$$

where the mask is applied to prevent the loss from being computed on categories for which an instance is not labeled. Once trained, the model was evaluated by picking the threshold that maximizes $F_{1}$ score on the validation set for each label and using these thresholds to predict labels for the seven classes. An aggregate label of 1 was assigned when all predicted classes were positive and 0 otherwise.

### 3.3 Zero-Shot Filtering

Zero-shot classification methods enable document filtering without requiring significant computational resources for model training or data labeling. Prior works have used natural language inference (NLI) based methods for zero-shot text classification by modeling it as a textual entailment task. Such models are trained to determine if one statement naturally follows from another.

We utilized an NLI-based method for zero-shot text classification by adapting pre-trained large language models such as BART [33], RoBERTa [35], XLM-RoBERTa [8], and DeBERTa [27], which were fine-tuned on NLI tasks.

For each label, we created a set of hypothesis templates, which are text statements indicating that an abstract did or did not meet the given inclusion criterion. Classification was performed by concatenating a document with the positive and negative hypothesis templates, passing it through the pre-trained model, and comparing the relative entailment probabilities of the positive and negative hypotheses. We experimented with multiple templates for each class, and the best-performing templates are given in Table 4.

The training data was used only to determine the optimal probability threshold for classifying an input as the positive class. This threshold is selected by computing the precision-recall curve and selecting the threshold where precision is equal to recall on the training data. This threshold is then fixed for evaluation on the test data. Predictions were made separately for each label. An aggregate label of 1 was assigned when all class-wise labels were 1.

### 3.4 Active Learning

Labeling documents for drug repurposing is a complex task requiring a certain level of medical expertise, making documents more difficult and expensive to label. Active learning (AL) proposes to iteratively select the most informative unlabeled instances for human labeling based on a mathematical query strategy. Newly labeled data is then used to update the model, and the process repeats until a stopping criterion is met. This process aims to maximize model performance given a limited labeling annotation budget. In theory, this process allows for the annotation of a smaller volume of data to achieve a similar level of predictive quality.

For our study, we used AL to finetune PubMedBERT [25] and compared three well-known query strategies described in a recent review by Schroeder et al. [51] along with a random sampling baseline. Query strategies used a pool-based approach, where a batch of k samples is selected for annotation at each iteration. All query strategies used implementations from the small-text AL library [52] with batches of k=20 samples.

For our query strategies, we denote instances by $x_{1},x_{2},\ldots ,x_{n}$, and the respective label for each instance $x_{i}$ is $y_{i}$, where $\forall i,y_{i}\in 0, 1$. The predicted class distribution is denoted by $P\left(y_{i}|x_{i}\right)$. Our query strategies are as follows:

1. Random Sampling (RS) selects the samples uniformly from the unlabeled data pool. This is the most commonly used baseline against which other query strategies are compared.
2. Prediction Entropy (PE) [48, 50] selects unlabeled samples highest entropy to minimize the overall entropy.
(5) $$\underset{x_{i}}{\mathrm{argmax}}\left[-\sum_{j=0}^{1}P\left(y_{i}=j|x_{i}\right)\log\left(P\left(y_{i}=j|x_{i}\right)\right)\right]$$
3. Least Confidence (LC) [9] picks the sample whose top prediction $k^{*}$ from the current model has the least confidence.
(6) $$\underset{x_{i}}{\mathrm{argmax}}\left[1-P\left(y_{i}=k_{1}^{*}|x_{i}\right)\right]$$
4. Breaking Ties (BT) [36, 49] takes the samples with the minimum gap between the top two most likely probabilities.
(7) $$\underset{x_{i}}{\mathrm{argmin}}\left[P\left(y_{i}=k_{1}^{*}|x_{i}\right)-P\left(y_{i}=k_{2}^{*}|x_{i}\right)\right]$$
  where $k_{1}^{*}$ is the most likely label and $k_{2}^{*}$ is the second most likely label.

We evaluated all the above query strategies for seven labels separately and classified the aggregate label as 1 if all the seven labels are 1 otherwise, 0.

### 3.5 Supervised Learning

Given the performance of large, transformer-based language models on document classification, we fine-tuned a diverse collection of biomedical language models on BioSift. All models were finetuned for 5 epochs with a batch size of 16 and weight decay of 0.01. The model from the best-performing epoch (as determined by the validation set) was evaluated on the test set at the end of training. Models included are PubMedBERT [25], BioBERT [31], RoBERTa [35], KRISSBERT [58], SapBERT [34], BART [33], BigBird [57], and BioELECTRA [28].

## 4 RESULTS & DISCUSSION

### 4.1 Overall Results

The results of all tested models’ ability to predict the multi-class labels of BioSift are shown in Table 5.

Fully supervised transformer models outperform other low-resource strategies for predicting each individual label and the aggregate document label.

Weakly supervised models have high recall but low precision. This result is likely due to the high propensity of LFs to label positive, which exaggerates the class imbalance beyond what is actually present in the dataset. Thus, weak supervision tends to under-filter documents for drug repurposing.

AL methods generally have lower recall than methods that learn from more samples. Here, the AL methods are often more precise than other low-resource methods but are more likely to miss documents with positive labels that should be included for drug repurposing.

PubMed filters tend to be more precise than other filtering metrics, sometimes even exceeding fully-supervised precision. PubMed often excludes a more significant proportion of documents that should be included for drug repurposing.

Our overall results illustrate that document filtering for drug repurposing is a very challenging task. Despite being widely known for inefficiently filtering abstracts for drug repurposing, carefully crafted PubMed queries often outperform the filtering ability of state-of-the-art low-resource machine learning algorithms. Our results highlight the need for new algorithms to improve the accuracy of document filtering tasks for drug repurposing.

### 4.2 Comparison with PubMed Filtering

PubMed advanced search filtering is the primary method biomedical researchers use to identify and select relevant abstracts for a particular research area. For each category annotated in our dataset, we used multiple advanced queries to replicate the results in our annotated dataset. Table 6 shows the PubMed filtering arguments that produced the best $F_{1}$ score for each category. While some PubMed filters can be quite precise, they often omit large numbers of documents that would be otherwise desirable to include in a meta-analysis. Notably, each PubMed filter can throw out up to 40% of results with each desirable property, which compounds with aggregation. Moreover, PubMed does not provide any means of filtering for drug/disease focused studies beyond the MeSH terms included in our initial query.

Table 7 gives examples of documents that were incorrectly included. Here, keyword-based PubMed searches fail to filter out abstracts that do not meet inclusion criteria. Similarly, Table 8 shows documents incorrectly excluded based on PubMed filtering. Here, very clear examples of clinical trials with carefully delineated comparator groups and quantified results were removed that should have been included.

### 4.3 Weakly Supervised Learning Results

Weak supervision has the potential to make learning significantly more efficient by reducing the need for annotators to label abstracts individually. We evaluate the extent to which weak supervision can label each class by post hoc computation of coverage, precision, recall, and other metrics on the train set of BioSift. These results are summarized in Table 9. LF evaluation shows substantial disparities in coverage between classes, with Cohort Study/Clinical Trial and Comparator Group having the lowest coverage, and Study Drug, Target Disease, and Human Subjects having the highest coverage. We also see that majority voting consistently outperforms the Snorkel label model by a small margin. This may be due to the large class imbalance present in the LF outputs due to the difficulty of creating exclusion rules.

### 4.4 Utility of Active Learning

Due to the relatively high cost of annotating examples in the biomedical domain, we evaluate whether active learning can be used to annotate a smaller pool of abstracts while achieving comparable accuracy. The active learning section of Table 5 shows that the best AL method with 50 query batches (1,000 total samples) has better precision than weak supervision but lags behind all other models in recall.

**Figure 7:**
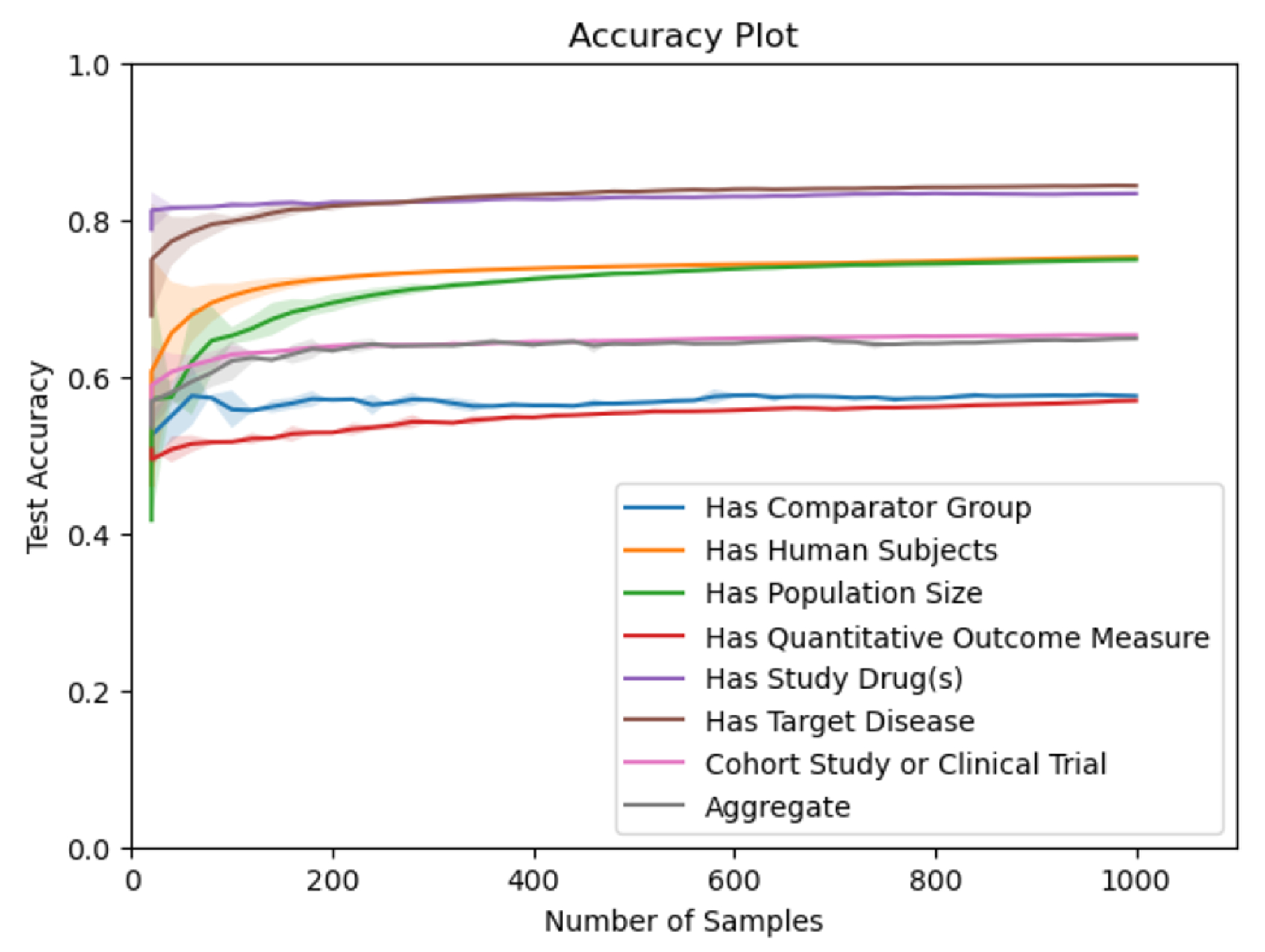
Classification Accuracy vs. Number of AL Samples

We also evaluated how much each AL model continues to improve model performance as the total number of samples increases. Figure 7 shows accuracy vs. number samples for prediction entropy, the query strategy with the highest $F_{1}$ score. This figure illustrates that model performance rapidly improves near the beginning of training but slows considerably for most classes between 200 and 400 samples.

## 5 RELATED WORK

### 5.1 NLP Drug Repurposing & Meta-Analysis

Natural language processing has recently shown strong potential for synthesizing evidence for systematic reviews of biomedical literature [1, 38]. However, these reviews rely upon PubMed filtering to select data articles to be included in such reviews [4, 53]. This results in systems that are either highly restrictive in the types of evidence that can be included or that require further manual curation or rule-based filtering [12, 38]. While some published works construct filtering datasets for specific diseases such as cancer [4], the developed datasets are proprietary and not accessible for use by the general research community. BioSift makes this task more accessible by open-sourcing such data for public, unrestricted use.

A few recent datasets seek to enable the extraction of PICO elements from clinical trials to facilitate evidence-based medicine. Nye et al. [43] use crowd workers to provide detailed annotations of patients, interventions, and outcomes in a corpus of clinical trials. Similarly, Zlabinger et al. develop a PICO annotation protocol that leads to improved annotation outcomes and use this to present an additional corpus with token-level PICO tags. Thomas et al. [54] develops a machine learning model for classifying whether or not a clinical study is a randomized controlled trial. BioSift complements these projects in enabling researchers to filter based on additional inclusion criteria to facilitate the automation of medical evidence synthesis.

### 5.2 Weakly Supervised Learning

Dua et al. [12] build a weakly supervised pipeline to filter documents for repurposing non-cancer drugs for cancer treatment. The authors develop a set of labeling functions targeted at excluding abstracts that are about cancer-related genes, cancer prevention, and premalignant patients. Similar to our weak supervision sources, they also create LFs using SciSpacy to determine if relevant diseases and drugs are present in documents. However, BioSift presents LFs aimed at a more general goal and provides an open-source resource for the development and evaluation of weak supervision for drug repurposing, which Dua et al. do not.

Dhrangadhariya and Müller develop a weak supervision pipeline for recognizing token-level PICO elements in text using expertdefined heuristics and alias matching to biomedical ontologies. BioSift differs from their work by presenting a new dataset and focusing on document filtering instead of token classification.

### 5.3 Zero-Shot Filtering

Yin et al. [56] first propose approaching zero-shot text classification as a textual entailment problem. They train a BERT[10] model on mainstream entailment datasets to learn the relationships between premises and hypotheses. For zero-shot classification, they convert labels into hypotheses and then use the previously pre-trained model to get an entailment decision.

In the biomedical domain, Barker et al. [5] propose a hybrid architecture that pairs a supervised text classification model with an NLI reranker to improve classification performance when training data is abundant for some classes but scarce or even nonexistent for others. Koutsomitropoulos [29] also suggests validating the quality of ontology-based annotations of biomedical resources using NLI models such as BART [33] and XLM-R [8], to overcome training barriers posed by large label sets and scarcity of data.

### 5.4 Active Learning

Active learning was first introduced by David and Gale [32], where they introduced uncertainty sampling to text classification. They iteratively sample low-confidence examples for labeling until a target accuracy is reached. In the biomedical domain, Guo et al. [26] used SVM-based active learning to annotate biomedical articles and achieved 82% accuracy with 2% of the examples used to train a similar fully supervised model. Active learning is frequently used in annotation pipelines to accelerate the work of human labelers [42] and is a common component of many commercial annotation platforms [44, 45].

## 6 CONCLUSION

This paper presents a new, original document classification dataset, BioSift, consisting of 10,000 human-annotated abstracts to expedite the initial selection and labeling of studies for drug repurposing. Each abstract is annotated by at least three human annotators and undergoes subsequent quality control. Robust benchmark results on the dataset illustrate neither PubMed advanced filters nor stateof-the-art document classification algorithms can efficiently replace human annotation. Thus, the publicly available dataset, BioSift, facilitates the future development of improved algorithms for document filtering aimed at drug repurposing.

## Figures and Tables

**Table 1:** Dataset Statistics

Total documents	10,000
Avg. words/doc	253.6
Avg. substances/doc	4.62
Year range	1969 - 2022

Has Human Subjects	9,337
Has Target Disease	9,316
Cohort Study or Clinical Trial	8,898
Has Quantitative Outcome	6,913
Has Study Drug	9,276
Has Population Size	8,698
Has Comparator Group	5,255

**Table 2:** Examples of included drugs in pre-filtering PubMed queries for cancer drug repurposing

Comorbid Condition	Drug Example(s)

Diabetes	Metformin
Hypertension	Lisinopril, Beta-blockers
Asthma	Adrenergic beta agonists
Hypothyroidism	Levothyroxine
Sleep disorders	Zolpidem
Neuropathy	Gabapentin
Hyperlipidemia	Atorvastatin, Simvastatin
Depression	Fluoxetine

**Table 3:** Labeling functions for each category

Class	Rules
**Has Comparator**	INCLUDE [“control group”, “placebo”, “compared to/with control”, “double/single blind”, “group A”, “intervention arm”]
**Cohort Study / Clinical Trial**	INCLUDE [“randomized controlled trial(s)”, “clinical trial(s)”, “cohort study(s)”], EXCLUDE “meta analysis”
**Has Pop. Size**	INCLUDE [“[number]” + max of 20 chars + “patients”, “n = [number]”, “population size”, “sample size”, “[number]” + “volunteers”, “[number]” + “subjects”]
**Has Quant. Outcome**	INCLUDE [p-val, OR, CI, HR, RR], EXCLUDE lack of any number
**Has Human Subjects**	INCLUDE [“hospital stay”, “admission”, “discharge”, “subjects”, “participants”, “volunteers”, “patients”], EXCLUDE [“rats”, “mice”]
**Has Study Drug**	INCLUDE compare with list of FDA approved drugs, “study drug(s)”, EXCLUDE if scispacy’s en_ner_-bc5cdr_md cannot detect entities of type CHEMICAL
**Has Target Disease**	INCLUDE “disease”, “cancer”, EXCLUDE if scispacy’s en_ner_bc5cdr_md cannot detect entities of type DISEASE

**Table 4:** Hypothesis template and candidate labels used for each of the 7 tasks on Zero-Shot Learning

	Hypothesis template: “This study {}.”	
Target label	Positive candidate	Negative candidate

**Cohort Study / Clinical Trial**	has a cohort study or clinical trial	does not have any cohorts or clinical trial
**Has Comparator Group**	has a control, double-blind, or comparison patient group	does not have any comparison patient group
**Has Human Subjects**	has human subjects	does not have human subjects
**Has Population Size**	contains population size or sample size information	does not contain population size information
**Has Quant. Outcome**	has quantitative outcomes like numbers, P-value, OR, CI, HR, RR, or patient ratios	does not have any quantitative outcomes
**Has Study Drug(s)**	has a target drug	does not have a target drug
**Has Target Disease**	has a target disease	does not have a target disease

**Table 5:** Multi-label Classification Results

	Model	Aggregate			Cohort/Clinical Study			Comparator Group			Human Subjects			Population Size			Quantitative Outcome			Study Drug			Target Disease		
		F1	P	R	F1	P	R	F1	P	R	F1	P	R	F1	P	R	F1	P	R	F1	P	R	F1	P	R
	PubMed Filtering	0.463	0.704	0.345	0.792	0.939	0.684	0.649	0.704	0.602	0.950	0.960	0.941	0.861	0.897	0.828	0.847	0.977	0.748	-	-	-	-	-	-

Weak Supervision	BART [22]	0.498	0.348	0.868	0.942	0.890	1.000	0.749	0.709	0.794	0.972	0.948	0.997	0.943	0.923	0.965	0.831	0.714	0.993	0.966	0.935	1.000	0.974	0.961	0.987
	RoBERTa [23]	0.500	0.351	0.871	0.942	0.890	1.000	0.740	0.730	0.751	0.970	0.946	0.996	0.936	0.880	1.000	0.831	0.727	0.970	0.967	0.936	1.000	0.971	0.945	0.999
	KRISSBERT [19]	0.501	0.341	0.944	0.943	0.893	0.999	0.727	0.644	0.836	0.971	0.946	0.997	0.945	0.922	0.969	0.842	0.756	0.949	0.966	0.935	1.000	0.972	0.960	0.985
	BioBERT [21]	0.503	0.342	**0.954**	0.944	0.896	0.998	0.763	0.724	0.806	0.973	0.947	1.000	0.942	0.905	0.982	0.830	0.725	0.971	0.966	0.935	1.000	0.974	0.956	0.994
	BlueBERT [13]	0.511	0.387	0.749	0.945	0.898	0.997	0.774	0.732	0.823	0.973	0.947	1.000	0.937	0.886	0.994	0.834	0.749	0.941	0.966	0.934	1.000	0.974	0.958	0.990
	PubMedBERT [20]	0.515	0.371	0.838	0.943	0.895	0.997	0.753	0.702	0.813	0.971	0.943	1.000	0.939	0.927	0.951	0.861	0.792	0.942	0.967	0.938	0.998	0.974	0.963	0.985
	SapBERT [14]	0.528	0.397	0.785	0.944	0.894	0.999	0.746	0.740	0.753	0.973	0.947	0.999	0.938	0.903	0.975	0.831	0.711	1.000	0.967	0.934	0.998	0.973	0.948	0.999
	BioELECTRA [18]	**0.537**	**0.403**	0.805	0.942	0.890	1.000	0.738	0.775	0.704	0.972	0.946	0.999	0.945	0.915	0.977	0.862	0.787	0.952	0.967	0.937	0.999	0.973	0.947	0.999

Zero-Shot	XLM-RoBERTa [17]	0.409	0.344	0.505	0.900	0.897	0.903	0.577	0.585	0.570	0.947	0.953	0.941	0.887	0.891	0.884	1.696	0.691	0.702	0.935	0.939	0.930	0.943	0.946	0.939
	DeBERTA (NLI) [30]	0.413	0.410	0.416	0.891	0.895	0.886	0.664	0.682	0.647	0.945	0.951	0.939	0.893	0.900	0.886	0.775	0.778	0.771	0.934	0.939	0.928	0.953	0.951	0.955
	ROBERTa (MNLI) [24]	0.420	0.341	0.548	0.893	0.894	0.892	0.584	0.585	0.583	0.952	0.955	0.950	0.870	0.876	0.863	0.717	0.713	0.721	0.941	0.940	0.942	0.940	0.944	0.935
	BART (MNLI) [15]	**0.549**	**0.534**	**0.564**	0.923	0.919	0.926	0.735	0.721	0.749	0.968	0.975	0.961	0.930	0.926	0.934	0.791	0.785	0.797	0.956	0.968	0.944	0.966	0.962	0.970

Active Lrng	PubMedBERT [25]-RS	0.258	0.462	0.225	0.947	0.915	0.982	0.529	0.420	0.901	0.856	0.746	0.972	0.946	0.947	0.936	0.816	0.756	0.874	0.937	0.987	0.882	0.972	0.973	0.961
	PubMedBERT [25]-LC	0.301	**0.491**	0.175	0.635	0.951	0.472	0.646	0.649	0.636	0.680	0.959	0.527	0.738	0.892	0.621	0.526	0.674	0.435	0.758	0.971	0.625	0.651	0.962	0.491
	PubMedBERT [25]-BT	0.314	0.476	0.267	0.846	0.935	0.773	0.697	0.642	0.749	0.586	0.941	0.429	0.658	0.875	0.528	0.746	0.705	0.791	0.701	0.982	0.556	0.824	0.951	0.735
	PubMedBERT [25]-PE	**0.446**	0.452	**0.435**	0.799	0.679	0.976	0.535	0.389	0.866	0.863	0.758	0.989	0.871	0.793	0.983	0.723	0.596	0.946	0.937	0.897	0.977	0.921	0.864	0.980

Supervised Learning	BigBird [16]	0.634	0.612	0.657	0.950	0.922	0.980	0.766	0.759	0.773	0.968	0.950	0.988	0.954	0.929	0.981	0.859	0.828	0.893	0.965	0.933	0.999	0.970	0.943	0.998
	BioBERT [21]	0.646	0.605	0.693	0.949	0.916	0.984	0.783	0.775	0.791	0.978	0.969	0.986	0.972	0.962	0.982	0.864	0.828	0.904	0.971	0.954	0.990	0.974	0.957	0.992
	RoBERTa [23]	0.653	0.588	0.735	0.950	0.914	0.988	0.774	0.749	0.801	0.980	0.967	0.993	0.978	0.975	0.982	0.089	0.846	0.893	0.966	0.946	0.987	0.971	0.947	0.997
	BART [22]	0.658	0.585	0.753	0.950	0.920	0.982	0.797	0.757	0.843	0.981	0.971	0.991	0.976	0.964	0.988	‘0.881	0.847	0.918	0.966	0.944	0.989	0.974	0.955	0.993
	KRISSBERT [19]	0.677	0.597	0.781	0.949	0.913	0.987	0.805	0.774	0.839	0.983	0.971	0.994	0.983	0.976	0.990	0.888	0.852	0.927	0.972	0.947	0.998	0.972	0.953	0.992
	SapBERT [14]	0.681	0.624	0.749	0.950	0.921	0.981	0.808	0.780	0.839	0.983	0.974	0.992	0.980	0.968	0.993	0.890	0.874	0.907	0.968	0.947	0.991	0.973	0.959	0.986
	BioELECTRA [18]	0.682	**0.636**	0.735	0.950	0.912	0.992	0.802	0.793	0.811	0.973	0.956	0.991	0.975	0.961	0.990	0.894	0.878	0.912	0.965	0.933	1.000	0.970	0.941	1.000
	PubMedBERT [20]	**0.696**	0.620	**0.792**	0.947	0.913	0.984	0.806	0.762	0.855	0.983	0.974	0.991	0.987	0.982	0.992	0.898	0.879	0.919	0.971	0.952	0.991	0.974	0.960	0.989

**Table 6:** Best performing PubMed Advanced Search arguments

Category	Pos. Ratio	F1	P	R	Best Filtering Args
**Has Comparator**	0.521	0.649	0.704	0.602	AND (“control”[All Fields] OR “comparator”[All Fields] OR “double blind”[All Fields] OR “double-blind”[All Fields] OR “study arm”[All Fields])
**Cohort Study / Clinical Trial**	0.887	0.792	0.939	0.684	AND (clinicalstudy[Filter] OR clinicaltrial[Filter] OR controlledclinicaltrial[Filter] OR multi-centerstudy[Filter] OR observationalstudy[Filter] OR randomizedcontrolledtrial[Filter])
**Quant. Outcome**	0.690	0.847	0.977	0.748	AND (“odds ratio”[Title/Abstract] OR “hazard ratio”[Title/Abstract] OR “p =“[Title/Abstract] OR “95% CI”[Title/Abstract] OR “risk ratio”[Title/Abstract])
**Has Pop. Size**	0.869	0.861	0.897	0.828	AND “patients”[Title/Abstract]
**Human Subjects**	0.932	0.971	0.946	0.997	AND (humans[Filter])
**Has Study Drug**	0.928	-	-	-	-
**Has Target Disease**	0.933	-	-	-	-

**Table 7:** False Positives produced by PubMed search

Class	PMID	Reason for Exclusion
**Has Comparator**	34822104	Study does not describe any patient treatment/comparator groups.
	6108780	Clinical trial has a single group of patients with no comparison.

**Cohort Study / Clinical Trial**	8198018	Describes biopharmaceutical properties of fluvastatin; no study done in patient popluation.
	13129875	Study design is a “retrospective, noncomparative, interventional case series.”

**Has Pop. Size**	6369972	Does not mention a number of patients.
	19897698	Review paper; does not list number of patients.

**Has Quant. Outcome**	31258919	Does not quantify study outcomes in abstract.
	8877074	Comparison of elanopril and losartan is not explicitly quantified.

**Has Human Subjects**	7015670	Does not explicitly identify humans in discussion of cinoxacin.
	31142401	This study is an animal model in prarie dogs.

**Table 8:** Articles incorrectly excluded by PubMed filtering

Class	PMID	Evidence for Inclusion
**Has Comparator**	32506444	“…we enrolled 708 patients with ACS treated with clopidogrel (n=137), ticagrelor (n=260) or prasugrel (n=311)…”
	33439469	“…Patients were divided into two uric acid categories, with uric acid ≤ 0.36 mmol/L and > 0.36 mmol/L…”

**Cohort Study / Clinical Trial**	25857447	“…medical charts of 59 patients with total loss of hearing, defined as pure tone thresholds in the profound range (> 90 dB) with an unobtainable speech reception threshold (SRT) that were treated with OP (n=20), ITD (n=13), or OP followed by salvage ITD (n=26) were analyzed…
	12772798	“134 patients tested for Helicobacter pylori infection were infected, and 65/66 (98%) had inflammation…”

**Has Pop. Size**	10513459	“…Thirty-one children with ADHD participated in a double-blind crossover study…”
	27824554	“…We compared behavioral performance in 58 healthy humans treated during 8 weeks with either placebo or the selective serotonin reuptake inhibitor escitalopram…”

**Has Quant. Outcome**	8688757	“…Simvastatin reduced total cholesterol by 1.9 mmol/l (26.7%) at the time of follow up…”
	16358864	“…totally cured patients with (A+S) is 3.4% better that cured only with antibiotics in the same time…”

**Has Human Subjects**	7105533	“…this study was performed on a relatively small number of patients undergoing total hip arthroplasty…”
	7297143	“…We gave intravenously both 0.4 mg pindolol and placebo to 24 mild to moderate asthmatic subjects in remission…”

**Table 9:** Label Model and Majority Voter Performance

Metric	Cohort Study		Comp. Group		Human Subjects		Population Size		Quant. Outcome		Study Drug(s)		Target Disease	
	MV	LM	MV	LM	MV	LM	MV	LM	MV	LM	MV	LM	MV	LM
Accuracy	0.890	0.869	0.884	0.861	0.969	0.964	0.965	0.864	0.972	0.972	0.938	0.936	0.887	0.884
F1 Score	0.941	0.930	0.939	0.925	0.984	0.982	0.982	0.926	0.985	0.985	0.968	0.967	0.935	0.933
Precision	0.899	0.869	0.885	0.884	0.969	0.969	0.965	0.968	0.971	0.971	0.957	0.957	0.988	0.988
Recall	0.987	1.00	1.00	0.969	1.00	0.995	1.00	0.888	1.00	1.00	0.979	0.976	0.886	0.883
Coverage	0.173	0.176	0.331	0.331	0.898	0.902	0.751	0.751	0.465	0.466	0.944	0.946	0.320	0.321
False Positive Rate	0.131	0.098	0.112	0.115	0.031	0.031	0.028	0.035	0.028	0.028	0.041	0.041	0.009	0.008
False Negative Rate	0.0	0.012	0.027	0.0	0.004	0.0	0.108	0.0	0.0	0.0	0.022	0.020	0.106	0.103
False Abstain Rate	0.736	0.736	0.238	0.238	0.069	0.073	0.155	0.155	0.256	0.256	0.040	0.042	0.649	0.650

## Data Availability

BioSift is publicly available on GitHub: https://github.com/pathology-dynamics/biosift/. It will also be uploaded to the Hugging Face Hub.

## CCS CONCEPTS

Applied computing → Life and medical sciences; ⋅ Information systems → Information retrieval; Document filtering.

## References

[1] Al-Hussaini Irfan, Nakajima An Davi, Lee Albert J., Bi Sarah, and Mitchell Cassie S.. 2022. CCS Explorer: Relevance Prediction, Extractive Summarization, and Named Entity Recognition from Clinical Cohort Studies. In 2022 IEEE International Conference on Big Data (Big Data). 5173–5181. 10.1109/BigData55660.2022.10020807

[2] Ashburn Ted T and Thor Karl B. 2004. Drug repositioning: identifying and developing new uses for existing drugs. Nature reviews Drug discovery 3, 8 (2004), 673–683. 15286734 10.1038/nrd1468

[3] Bakowski Malina A, Beutler Nathan, Wolff Karen C, Kirkpatrick Melanie G, Chen Emily, Nguyen Tu-Trinh H, Riva Laura, Shaabani Namir, Parren Mara, Ricketts James, 2021. Drug repurposing screens identify chemical entities for the development of COVID-19 interventions. Nature communications 12, 1 (2021), 3309. 10.1038/s41467-021-23328-0 PMC8175350 34083527

[4] Baldini Ioana, Bernagozzi Mariana, Aggarwal Sulbha, Bornea Mihaela, Chawla Saksham, Geluykens Joppe, Katz-Rogozhnikov Dmitriy A, Mukherjee Pratik, Ramesh Smruthi, Rosenthal Sara, 2021. Exploring the efficacy of generic drugs in treating cancer. In Proceedings of the AAAI Conference on Artificial Intelligence, Vol. 35. 15988–15990.

[5] Barker Ken, Awasthy Parul, Ni Jian, and Florian Radu. 2021. IBM MNLP IE at CASE 2021 Task 2: NLI Reranking for Zero-Shot Text Classification. In Proceedings of the 4th Workshop on Challenges and Applications of Automated Extraction of Sociopolitical Events from Text (CASE 2021). Association for Computational Linguistics, Online, 193–202. 10.18653/v1/2021.case-1.24

[6] Bastian Hilda, Glasziou Paul, and Chalmers Iain. 2010. Seventy-five trials and eleven systematic reviews a day: how will we ever keep up? PLoS medicine 7, 9 (2010), e1000326. 20877712 10.1371/journal.pmed.1000326 PMC2943439

[7] Chong Curtis Rand Sullivan David J Jr. 2007. New uses for old drugs. Nature 448, 7154 (2007), 645–646. 17687303 10.1038/448645a

[8] Conneau Alexis, Khandelwal Kartikay, Goyal Naman, Chaudhary Vishrav, Wenzek Guillaume, Guzmán Francisco, Grave Edouard, Ott Myle, Zettlemoyer Luke and Stoyanov Veselin. 2020. Unsupervised Cross-lingual Representation Learning at Scale. In Proceedings of the 58th Annual Meeting of the Association for Computational Linguistics. Association for Computational Linguistics, Online, 8440–8451 10.18653/v1/2020.acl-main.747

[9] Culotta Aron and McCallum Andrew. 2005. Reducing Labeling Effort for Structured Prediction Tasks. In Proceedings of the 20th National Conference on Artificial Intelligence - Volume 2 (Pittsburgh, Pennsylvania) (AAAI’05). AAAI Press, 746–751.

[10] Devlin Jacob, Chang Ming-Wei, Lee Kenton, and Toutanova Kristina. 2019. BERT: Pre-training of Deep Bidirectional Transformers for Language Understanding. In Proceedings of the 2019 Conference of the North American Chapter of the Association for Computational Linguistics: Human Language Technologies, Volume 1 (Long and Short Papers). 4171–4186.

[11] Dickersin Kay, Scherer Roberta, and Lefebvre Carol. 1994. Systematic reviews: identifying relevant studies for systematic reviews. Bmj 309, 6964 (1994), 1286–1291. 7718048 10.1136/bmj.309.6964.1286 PMC2541778

[12] Dua Sejal, Baldini Ioana, Katz-Rogozhnikov Dmitriy A., Veen Emily van der, Britt Allison, Mangalath Pradeep, Kleiman Laura B., and Vecchio Fitz Catherine Del. 2021. Biomedical Corpus Filtering: A Weak Supervision Paradigm With Infused Domain Expertise. In SDU@AAAI.

[13] Hugging Face. 2023. bionlp/bluebert_pubmed_mimic_uncased_L-12_H-768_A-12. https://huggingface.co/bionlp/bluebert_pubmed_mimic_uncased_L-12_H-768_A-12

[14] Hugging Face. 2023. cambridgeltl/SapBERT-from-PubMedBERT-fulltext. https://huggingface.co/cambridgeltl/SapBERT-from-PubMedBERT-fulltext

[15] Hugging Face. 2023. facebook/bart-large-mnli. https://huggingface.co/facebook/bart-large-mnli

[16] Hugging Face. 2023. google/bigbird-pegasus-large-pubmed. https://huggingface.co/google/bigbird-pegasus-large-pubmed

[17] Hugging Face. 2023. joeddav/xlm-roberta-large-xnli. https://huggingface.co/joeddav/xlm-roberta-large-xnli

[18] Hugging Face. 2023. kamalkraj/bioelectra-base-discriminator-pubmed. https://huggingface.co/kamalkraj/bioelectra-base-discriminator-pubmed

[19] Hugging Face. 2023. microsoft/BiomedNLP-KRISSBERT-PubMed-UMLS-EL. https://huggingface.co/microsoft/BiomedNLP-KRISSBERT-PubMed-UMLS-EL

[20] Hugging Face. 2023. microsoft/BiomedNLP-PubMedBERT-base-uncased-abstract-fulltext. https://huggingface.co/microsoft/BiomedNLP-PubMedBERT-base-uncased-abstract-fulltext

[21] Hugging Face. 2023. monologg/biobert_v1.1_pubmed. https://huggingface.co/monologg/biobert_v1.1_pubmed

[22] Hugging Face. 2023. mse30/bart-base-finetuned-pubmed. https://huggingface.co/mse30/bart-base-finetuned-pubmed

[23] Hugging Face. 2023. raynardj/ner-gene-dna-rna-jnlpba-pubmed. https://huggingface.co/raynardj/ner-gene-dna-rna-jnlpba-pubmed

[24] Hugging Face. 2023. roberta-large-mnli. https://huggingface.co/roberta-large-mnli

[25] Gu Yu, Tinn Robert, Cheng Hao, Lucas Michael, Usuyama Naoto, Liu Xiaodong, Naumann Tristan, Gao Jianfeng, and Poon Hoifung. 2020. Domain-Specific Language Model Pretraining for Biomedical Natural Language Processing. arXiv:arXiv:2007.15779

[26] Guo Yufan, Silins Ilona, Stenius Ulla, and Korhonen Anna. 2013. Active learning-based information structure analysis of full scientific articles and two applications for biomedical literature review. Bioinformatics 29, 11 (June 2013), 1440–1447. 23564844 10.1093/bioinformatics/btt163

[27] He Pengcheng, Liu Xiaodong, Gao Jianfeng, and Chen Weizhu. 2020. DeBERTa: Decoding-enhanced BERT with Disentangled Attention. 10.48550/ARXIV.2006.03654

[28] Kanakarajan Kamal raj, Kundumani Bhuvana, and Sankarasubbu Malaikannan. 2021. BioELECTRA:Pretrained Biomedical text Encoder using Discriminators. In Proceedings of the 20th Workshop on Biomedical Language Processing. Association for Computational Linguistics, Online, 143–154. 10.18653/v1/2021.bionlp-1.16

[29] Koutsomitropoulos Dimitrios. 2021. Validating Ontology-based Annotations of Biomedical Resources using Zero-shot Learning. In The 12th International Conference on Computational Systems-Biology and Bioinformatics. 37–43.

[30] Laurer Moritz, Atteveldt Wouter van, Casas Andreu Salleras, and Welbers Kasper. 2022. Less Annotating, More Classifying – Addressing the Data Scarcity Issue of Supervised Machine Learning with Deep Transfer Learning and BERT-NLI. Preprint. https://osf.io/74b8k

[31] Lee Jinhyuk, Yoon Wonjin, Kim Sungdong, Kim Donghyeon, Kim Sunkyu, So Chan Ho, and Kang Jaewoo. 2020. BioBERT: a pre-trained biomedical language representation model for biomedical text mining. Bioinformatics 36, 4 (2020), 1234–1240. 31501885 10.1093/bioinformatics/btz682 PMC7703786

[32] Lewis David D. and Gale William A.. 1994. A Sequential Algorithm for Training Text Classifiers. 10.48550/ARXIV.CMP-LG/9407020

[33] Lewis Mike, Liu Yinhan, Goyal Naman, Ghazvininejad Marjan, Mohamed Abdelrahman, Levy Omer, Stoyanov Veselin, and Zettlemoyer Luke. 2020. BART: Denoising Sequence-to-Sequence Pre-training for Natural Language Generation, Translation, and Comprehension. In Proceedings of the 58th Annual Meeting of the Association for Computational Linguistics. Association for Computational Linguistics, Online, 7871–7880. 10.18653/v1/2020.acl-main.703

[34] Liu Fangyu, Shareghi Ehsan, Meng Zaiqiao, Basaldella Marco, and Collier Nigel. 2021. Self-Alignment Pretraining for Biomedical Entity Representations. In Proceedings of the 2021 Conference of the North American Chapter of the Association for Computational Linguistics: Human Language Technologies. Association for Computational Linguistics, Online, 4228–4238. https://www.aclweb.org/anthology/2021.naacl-main.334

[35] Liu Yinhan, Ott Myle, Goyal Naman, Du Jingfei, Joshi Mandar, Chen Danqi, Levy Omer, Lewis Mike, Zettlemoyer Luke, and Stoyanov Veselin. 2019. RoBERTa: A Robustly Optimized BERT Pretraining Approach. arXiv preprint arXiv:1907.11692 (2019).

[36] Luo Tong, Kramer Kurt, Goldgof Dmitry B., Hall Lawrence O., Samson Scott, Remsen Andrew, and Hopkins Thomas. 2005. Active Learning to Recognize Multiple Types of Plankton. Journal of Machine Learning Research 6, 20 (2005), 589–613. http://jmlr.org/papers/v6/luo05a.html

[37] Marshall Iain J, Noel-Storr Anna, Kuiper Joël, Thomas James, and Wallace Byron C. 2018. Machine learning for identifying randomized controlled trials: an evaluation and practitioner’s guide. Research synthesis methods 9, 4 (2018), 602–614 29314757 10.1002/jrsm.1287 PMC6030513

[38] Marshall Iain J, Trikalinos Thomas A, Soboczenski Frank, Yun Hye Sun, Kell Gregory, Marshall Rachel, and Wallace Byron C. 2022. In a pilot study, automated real-time systematic review updates were feasible, accurate, and work-saving. Journal of Clinical Epidemiology (2022). 10.1016/j.jclinepi.2022.08.013 36150548

[39] Masoudi-Sobhanzadeh Yosef, Omidi Yadollah, Amanlou Massoud, and Masoudi-Nejad Ali. 2020. Drug databases and their contributions to drug repurposing. Genomics 112, 2 (2020), 1087–1095 31226485 10.1016/j.ygeno.2019.06.021

[40] McCoy Kevin, Gudapati Sateesh, He Lawrence, Horlander Elaina, Kartchner David, Kulkarni Soham, Mehra Nidhi, Prakash Jayant, Thenot Helena, Vanga Sri Vivek, 2021. Biomedical text link prediction for drug discovery: a case study with COVID-19. Pharmaceutics 13, 6 (2021), 794 34073456 10.3390/pharmaceutics13060794 PMC8230210

[41] Mitchell Cassie S, Cates Ashlyn, Kim Renaid B, and Hollinger Sabrina K. 2015. Undergraduate biocuration: developing tomorrow’s researchers while mining today’s data. Journal of Undergraduate Neuroscience Education 14, 1 (2015), A56. 26557796 PMC4640483

[42] Monarch Robert Munro. 2021. Human-in-the-Loop Machine Learning: Active learning and annotation for human-centered AI. Simon and Schuster.

[43] Nye Benjamin, Li Junyi Jessy, Patel Roma, Yang Yinfei, Marshall Iain, Nenkova Ani, and Wallace Byron. 2018. A Corpus with Multi-Level Annotations of Patients, Interventions and Outcomes to Support Language Processing for Medical Literature. In Proceedings of the 56th Annual Meeting of the Association for Computational Linguistics (Volume 1: Long Papers). Association for Computational Linguistics, Melbourne, Australia, 197–207. 10.18653/v1/P18-1019 PMC6174533 30305770

[44] Pei Jiaxin, Ananthasubramaniam Aparna, Wang Xingyao, Zhou Naitian, Dedeloudis Apostolos, Sargent Jackson, and Jurgens David. 2022. POTATO: The Portable Text Annotation Tool. In Proceedings of the The 2022 Conference on Empirical Methods in Natural Language Processing: System Demonstrations. Association for Computational Linguistics, Abu Dhabi, UAE, 327–337. https://aclanthology.org/2022.emnlp-demos.33

[45] Perry Tal. 2021. LightTag: Text Annotation Platform. In Proceedings of the 2021 Conference on Empirical Methods in Natural Language Processing: System Demonstrations. Association for Computational Linguistics, Online and Punta Cana, Dominican Republic, 20–27. 10.18653/v1/2021.emnlp-demo.3

[46] Prasad Vinay and Mailankody Sham. 2017. Research and development spending to bring a single cancer drug to market and revenues after approval. JAMA internal medicine 177, 11 (2017), 1569–1575. 28892524 10.1001/jamainternmed.2017.3601 PMC5710275

[47] Ratner Alexander, Bach Stephen H., Ehrenberg Henry, Fries Jason, Wu Sen and Ré Christopher. 2017. Snorkel: Rapid Training Data Creation with Weak Supervision. Proc. VLDB Endow 11, 3 (nov 2017), 269–282. 10.14778/3157794.3157797 PMC5951191 29770249

[48] Roy Nicholas and Andrew McCallum. 2001. Toward Optimal Active Learning through Sampling Estimation of Error Reduction. In Proceedings of the Eighteenth International Conference on Machine Learning (ICML ‘01). Morgan Kaufmann Publishers Inc., San Francisco, CA, USA, 441–448.

[49] Scheffer Tobias, Decomain Christian, and Wrobel Stefan. 2001. Active Hidden Markov Models for Information Extraction. In International Symposium on Intelligent Data Analysis.

[50] Schohn Greg and Cohn David. 2000. Less is More: Active Learning with Support Vector Machines. In Proceedings of the Seventeenth International Conference on Machine Learning (ICML ‘00). Morgan Kaufmann Publishers Inc., San Francisco, CA, USA, 839–846.

[51] Christopher Schröder Andreas Niekler, and Potthast Martin. 2022. Revisiting Uncertainty-based Query Strategies for Active Learning with Transformers. In Findings of the Association for Computational Linguistics: ACL 2022. Association for Computational Linguistics, Dublin, Ireland, 2194–2203. 10.18653/v1/2022.findings-acl.172

[52] Schröder Christopher, Müller Lydia, Niekler Andreas, and Potthast Martin 2021. Small-Text: Active Learning for Text Classification in Python. arXiv:2107.10314 [cs.LG]

[53] Subramanian Shivashankar, Baldini Ioana, Ravichandran Sushma, Katz-Rogozhnikov Dmitriy A, Ramamurthy Karthikeyan Natesan, Sattigeri Prasanna, Varshney Kush R, Wang Annmarie, Mangalath Pradeep, and Kleiman Laura B. 2020. A natural language processing system for extracting evidence of drug repurposing from scientific publications. In Proceedings of the AAAI Conference on Artificial Intelligence, Vol. 34. 13369–13381.

[54] Thomas James, Steve McDonald Anna Noel-Storr, Shemilt Ian, Elliott Julian, Mavergames Chris, and Marshall Iain J.. 2021. Machine learning reduced workload with minimal risk of missing studies: development and evaluation of a randomized controlled trial classifier for Cochrane Reviews. Journal of Clinical Epidemiology 133 (2021), 140–151. 10.1016/j.jclinepi.2020.11.003 33171275 PMC8168828

[55] Wouters Olivier J, McKee Martin, and Luyten Jeroen. 2020. Estimated research and development investment needed to bring a new medicine to market, 2009-2018. Jama 323, 9 (2020), 844–853. 32125404 10.1001/jama.2020.1166 PMC7054832

[56] Yin Wenpeng, Hay Jamaal, and Roth Dan. 2019. Benchmarking Zero-shot Text Classification: Datasets, Evaluation and Entailment Approach. In Proceedings of the 2019 Conference on Empirical Methods in Natural Language Processing and the 9th International Joint Conference on Natural Language Processing (EMNLPIJCNLP). Association for Computational Linguistics, Hong Kong, China, 3914–3923. 10.18653/v1/D19-1404

[57] Zaheer Manzil, Guruganesh Guru, Dubey Kumar Avinava, Ainslie Joshua, Alberti Chris, Ontanon Santiago, Pham Philip, Ravula Anirudh, Wang Qifan, Yang Li, and Ahmed Amr. 2020. Big Bird: Transformers for Longer Sequences. In Advances in Neural Information Processing Systems, Larochelle H, Ranzato M, Hadsell R, Balcan MF, and Lin H (Eds.), Vol. 33. Curran Associates, Inc., 17283–17297. https://proceedings.neurips.cc/paper/2020/file/c8512d142a2d849725f31a9a7a361ab9-Paper.pdf

[58] Zhang Sheng, Cheng Hao, Vashishth Shikhar, Wong Cliff, Xiao Jinfeng, Liu Xiaodong, Naumann Tristan, Gao Jianfeng, and Poon Hoifung. 2022. Knowledge-Rich Self-Supervision for Biomedical Entity Linking. In Findings of the Association for Computational Linguistics: EMNLP 2022. Association for Computational Linguistics, Abu Dhabi, United Arab Emirates, 868–880. https://aclanthology.org/2022.findings-emnlp.61

